# Invitation to the International Medico-Scientific Exhibition

**Published:** 1890-05

**Authors:** 


### INVITATION TO THE INTERNATIONAL MEDICO-SCIENTIFIC EXHIBITION, BERLIN, AUGUST, 1890.

In connection with the Tenth International Medical Congress, to be held in Berlin, August 4th-9th 1890, there will be an International Medico-Scientific Exhibition.

The undersigned Committee of Organization has been authorized, by the representatives of the medical faculties and leading medical societies of the German Empire to make the preliminary arrangements. We therefore cordially invite all who may wish to exhibit or participate in the above Exhibition. All exhibits, however, to be of a scientific nature.

The exhibits expected will be as follows:

1. New or improved Scientific Instruments for Biological and Special Medical Purposes, including apparatus for Photography and Spectral Analysis pertaining to medicine.
2. New Pharmacological Chemica Substances and Preparations.
3. New Pharmaceutical Substances and Preparations.
4. New Food Preparations.
5. New or improved Instruments for internal and external medicine and allied specialties, including Electrotherapy.
6. Plans and Models (new) of Hospitals, Houses for reconvalescents, disinfection, and general Bath-houses.
7. New appliances, such as pertain to nursing the sick, including the methods of transportaticn, and baths for the sick.
8. Apparatus (new) for Hygienic Purposes.

The special committee on "Exhibition" consists of the following gentlemen: Commerzienrath, Paul Dorffel, H. Haensch, Director, Dr. J. F. Holtz, Director, Dr. L. Loeweaherz, Regierungsrath, Dr. J. Petri, H. Windler, and the Secretary General of the Committee of Organization. The names of the Associate Members of the Exhibition Committee, as well as the names of the

Heads of Departments, will be made known shortly, also the conditions for Exhibiters.

For applications for exhibits, and information, please address Dr. O. Lassar, Secretary-General, Bureau of the Tenth International Medical Congress, Berlin, N. W. Carlstrasse No. 19.

Please designate all mail matter relating to the Exhibition, "Exhibition Affairs," and also enclose a visiting card, or card of the firm, on which the name and residence is plainly written or printed.

The Bureau is open for the present from 5-7 o'clock P. M.

THE COMMITTEE OF ORGANIZATION OF THE TENTH INTERNATIONAL MEDICAL CONGRESS.

Dr. Rudolf Virchow,
President.

Dr. E. von Bergmann, Dr. E. Leyden, Dr. W. Waldeyer,
Vice-Presidents.

Dr. O. Lassar,
Secretary-General.

---

### TENTH INTERNATIONAL MEDICAL CONGRESS.

